# Trajectories of Haemoglobin and incident stroke risk: a longitudinal cohort study

**DOI:** 10.1186/s12889-019-7752-7

**Published:** 2019-10-28

**Authors:** Alimu Dayimu, Wendi Qian, Bingbing Fan, Chunxia Wang, Jiangbing Li, Shukang Wang, Xiaokang Ji, Guangshuai Zhou, Tao Zhang, Fuzhong Xue

**Affiliations:** 1 Department of Biostatistics, School of Public Health, Shandong University, 44 Wenhuaxi Road, PO Box 100, Jinan, 250012 China; 2 Cambridge Clinical Trials Unit, Translational Research, University of Cambridge, Cambridge, England; 3 Health Management Center, Affiliated Hospital of Jining Medical University, Jining, Shandong China; 4 Department of Cardiology, Shandong Provincial Hospital, Shandong University, Jinan, Shandong China

**Keywords:** Haemoglobin, Stroke, Trajectory, Longitudinal cohort study

## Abstract

**Background:**

Studies have demonstrated that high or low haemoglobin increases the risk of stroke. Previous studies, however, performed only a limited number of haemoglobin measurements, while there are dynamic haemoglobin changes over the course of a lifetime. This longitudinal cohort study aimed to classify the long-term trajectory of haemoglobin and examine its association with stroke incidence.

**Methods:**

The cohort consisted of 11,431 participants (6549 men) aged 20 to 50 years whose haemoglobin was repeatedly measured 3–9 times during 2004–2015. A latent class growth mixture model (LCGMM) was used to classify the long-term trajectory of haemoglobin concentrations, and hazard ratios (HRs) and 95% confidence intervals (95% CI) according to the Cox proportional hazard model were used to investigate the association of haemoglobin trajectory types with the risk of stroke.

**Results:**

Three distinct trajectory types, high-stable (*n* = 5395), normal-stable (*n* = 5310), and decreasing (*n* = 726), were identified, with stroke incidence rates of 2.7, 1.9 and 3.2 per 1000 person-years, respectively. Compared to the normal-stable group, after adjusting for the baseline covariates, the decreasing group had a 2.94-fold (95% CI 1.22 to 7.06) increased risk of developing stroke. Strong evidence was observed in men, with an HR (95% CI) of 4.12 (1.50, 11.28), but not in women (HR = 1.66, 95% CI 0.34, 8.19). Individuals in the high-stable group had increased values of baseline covariates, but the adjusted HR (95% CI), at 1.23 (0.77, 1.97), was not significant for the study cohort or for men and women separately.

**Conclusions:**

This study revealed that a decreasing haemoglobin trajectory was associated with an increased risk of stroke in men. These findings suggest that long-term decreasing haemoglobin levels might increase the risk of stroke.

## Background

Studies have demonstrated that high or low haemoglobin levels are associated with an increased risk of developing stroke [1]. The association between low haemoglobin and the risk of stroke has been well studied [2]. However, studies have reported controversial results regarding the association between high haemoglobin concentration and the risk of stroke [1, 3–5]. A randomized controlled trial showed that treatment aimed at increasing the haemoglobin level doubled the risk of stroke [6]. Moreover, several studies reported that these effects only existed in women [4, 7], although men tended to have higher haemoglobin levels than women, and stroke was more common in men [8].

Most studies, however, focused on a single measurement of haemoglobin, ignoring the dynamic change of haemoglobin over the life-course. Haemoglobin concentration has been reported to be significantly associated with age and sex [9, 10]. A study reported that the longitudinal pattern of haemoglobin concentration after hospitalization was a better prognostic factor for heart failure than a single measurement [11]. To the best of our knowledge, no previous study has investigated the association of long-term haemoglobin trajectories with the risk of stroke in healthy adults.

This study included a longitudinal cohort from the Chinese population from 2004 to 2015. The aim was to identify the potential haemoglobin trajectory types and examine the association between haemoglobin trajectory types and incident stroke risk.

## Materials and methods

### Study cohort

The cohort data were collected from a population-based routine annual health check-up at the Centre of Health Management of Jining Medical University Hospital. Healthy adults underwent health examinations from May 2004 until September 2015. The health examination database reported various disease outcomes using a unique identification number for each participant [12]. All stroke events reported by the end of 2017 were included. The study included individuals aged between 20 and 50 years to exclude the potential impact of oestrogen deficiency after menopause on haemoglobin level and the risk of stroke in women, as 50 years is approximately the mean natural menopause age in China [13]. Participants without a previous history of stroke but with at least 3 haemoglobin measurements available were included. For individuals with reported stroke, all the data until the date of the first stroke were included. The participants were reimbursed by their employer for the health check-ups, so they rarely dropped out during the period of data collection.

Anonymous electronic records dataset was acquired from Jining Medical University Hospital. The study protocol was approved by the ethics committee of the School of Public Health, Shandong University.

### Examinations

Assessments of height, weight, systolic and diastolic blood pressures, smoking, alcohol consumption, haemoglobin, fasting blood glucose, lipids (total cholesterol, triglycerides, low-density lipoprotein cholesterol, high-density lipoprotein cholesterol), and creatinine were described previously [12]. The following definition of dyslipidaemia was based on the national cholesterol education programme adult treatment panel III criteria [14]: (1) total cholesterol > 6.20 mmol/L; (2) low-density lipoprotein cholesterol > 4.14 mmol/L; (3) high-density lipoprotein cholesterol < 1.04 mmol/L for men or < 1.30 mmol/L for women; and (4) triglycerides > 1.70 mmol/L. The estimated glomerular filtration rate (eGFR) was based on CKD-EPI (Chronic Kidney Disease Epidemiology Collaboration) equations [15]. Hypertension was defined as SBP/DBP ≥140/ 90 mmHg or diagnosis by medical records. Participants with fasting plasma glucose ≥7.0 mmol/L, HbA1c ≥ 6.5% or diagnosis by medical records were considered to have diabetes mellitus based on the Chinese guidelines for the prevention and treatment of diabetes (2013 edition).

### Outcome

The histories of stroke and stroke diagnosis were collected in the database, and the diagnostic date of stroke was defined as the earliest record date. We used the international classification of diseases 10th revision (ICD-10) clinical codes to identify cases. Subjects with ICD-10 codes from I60 to I69 were considered to have experienced stroke [16].

### Statistical analysis

Unsupervised cluster analysis using a latent class growth mixed model (LCGMM) was applied to explore the longitudinal heterogeneity in haemoglobin concentration. A series of polynomial specifications of haemoglobin as a function of age, with a class number ranging from 2 to 5, were assessed using the lcmm (version 1.7.9) package in R (version 3.5.0) [17]. The age of the participants was centred at the median age of the population and divided by 10 to reduce problems associated with high ages in quadratic and cubic terms in the models. We considered 3 possible polynomial specifications of the longitudinal response of haemoglobin as a function of age, linear, quadratic and cubic, to allow for non-linear patterns of haemoglobin in both fixed and random effect components. To address the sex differences in the haemoglobin trajectories, we also included a sex interaction with the polynomial function of age as a fixed effect in the model. For each model, we considered class-specific variance-covariance random-effects, which allowed for between-subjects’ trajectory variability to differ between classes. To avoid convergence towards local maxima, all models were rerun several times with different starting values and initial values obtained via grid searching (with a maximum of 15 iterations from 30 random vectors of values from the 1-class model).

The following criteria for choosing the best fit model together with the study specific requirements were used [17]: (1) a significant improvement of the model according to the Bayesian information criterion (BIC); (2) a posterior probability above 0.7; (3) a threshold of 0.65 for 70% or more of the participants in each class.

The characteristics across different groups were compared using analysis of variance or Kruskal-Wallis tests for continuous variables and χ^2^ statistics for categorical variables. The log-rank test was used for time-to-event variables. Cox proportional hazard models with follow-up time as the time scale were used to investigate the association between trajectory classes and incident stroke, with unadjusted (Model 1) or adjusted covariates (Model 2). The adjusted covariates included age at baseline, sex, smoking, alcohol consumption, GFR, BMI, diabetes mellitus, hypertension, and dyslipidaemia.

## Results

**Fig. 1:**
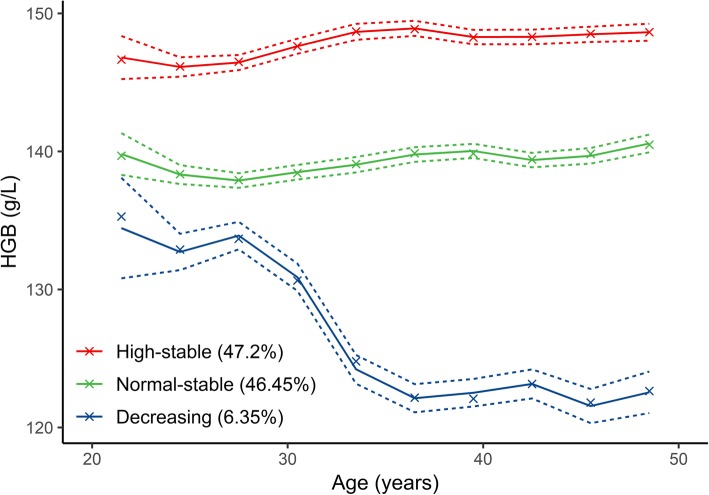
Estimated and observed mean trajectory of haemoglobin over age (crosses = estimated subject-specific mean trajectory; dashed line with dots = observed mean trajectory; dashed line = 95% confidence interval of the observed mean)

A total of 11,431 participants (6549 men) were included in this study. Additional file 1: Figure S1 and Table S1 show the flowchart of enrolment and a summary of the baseline characteristics of the included and excluded participants, respectively. The mean baseline age was 34.04 years (ranging from 20 to 48 years). On average, the participants’ haemoglobin was measured 4.0 times (SD 1.41, range 3–9). The median follow-up period was 4.4 years (SD 2.58, range 1.1–13.0), during which 134 strokes occurred; the incidence was 2.33 per 1000 person-years. Additional file 1: Table S2 presents the LCGMM results of the fitting process. A model of cubic parameters with three classes was chosen from all the investigated models according to the criteria mentioned above. Additional file 1: Table S3 shows the detailed parameter estimates of the best fitting three-class cubic trajectory model. Figure 1 shows the estimated conditional mean and observed mean trajectory of haemoglobin by age, with three trajectories: high-stable (47.2%, *n* = 5395), normal-stable (46.45%, *n* = 5310), decreasing (6.35%, *n* = 726). The estimated means were close to the observed means, suggesting a good fit of the model.

**Fig. 2:**
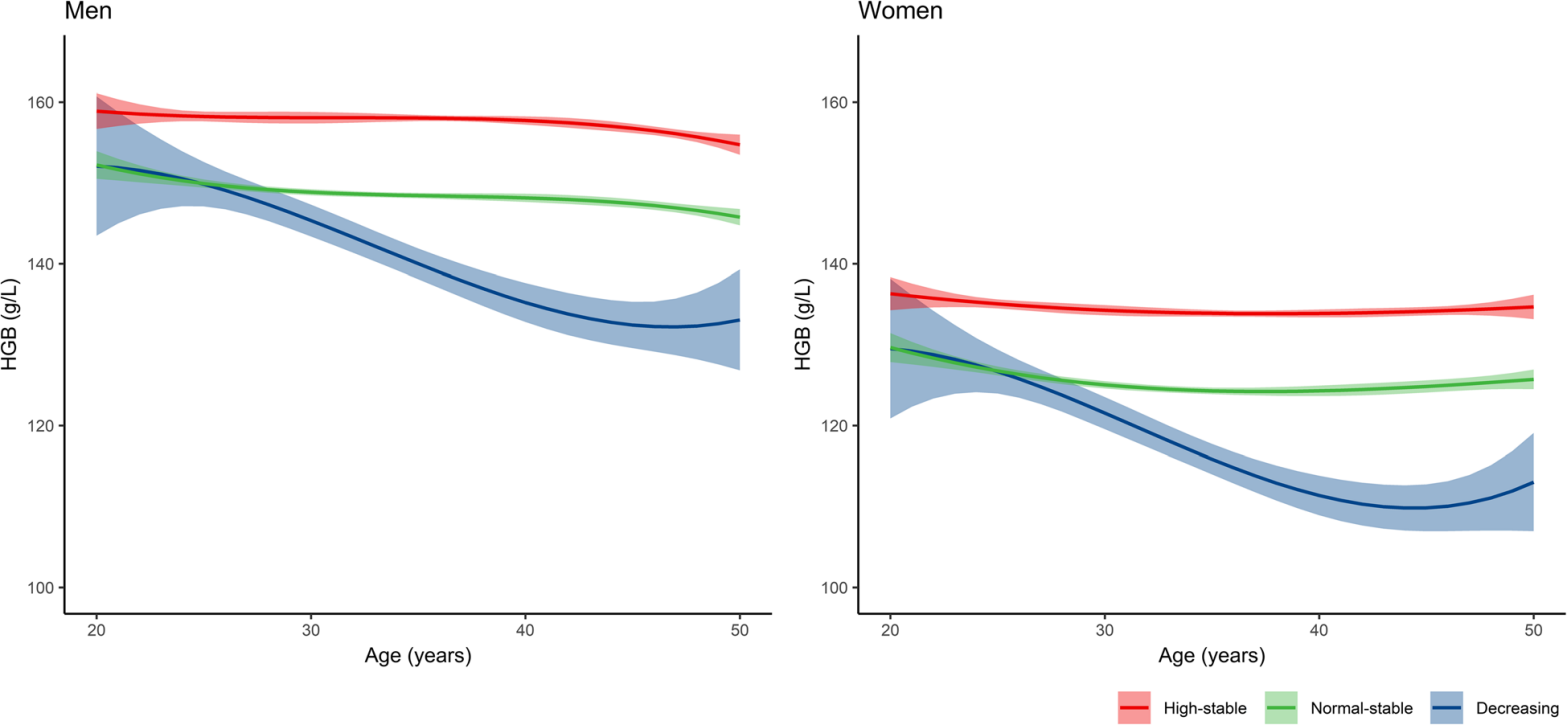
The predicted mean growth curves of three distinct haemoglobin trajectories for men and women. Solid lines show class-specific mean predicted levels as a function of age estimated from the best fitting growth mixture model (3-class cubic latent class growth mixture modelling), shaded areas indicate estimated 95% confidence intervals

Figure 2 shows the predicted mean trajectory of the three groups by sex**.** Except for a shift in the haemoglobin level, men and women had similar haemoglobin trajectory patterns by age. Haemoglobin in both the high-stable group and the normal-stable group slowly decreased with increasing age both in men and women, but a slow increasing trend was observed for women after the age of 40. In the decreasing group, the haemoglobin level persistently declined but slightly increased in men who were approximately 48 years old and in women who were approximately 45 years old.

Table 1 summarizes the baseline characteristics of the study population by haemoglobin trajectory types. The high-stable group had a higher baseline age, BMI, rate of hypertension, rate of dyslipidaemia, rate of smoking, and rate of alcohol consumption than the other groups. Most of the baseline characteristics were the lowest in the decreasing group, except for the eGFR and diabetes mellitus. In the normal-stable group, haemoglobin persisted at a normal level (mean = 138.1 g/L). In the high-stable group, haemoglobin persisted at a high level (mean = 149.0 g/L). Haemoglobin had the lowest concentration level but the highest standard deviation in the decreasing group. The incidence of stroke was higher in the decreasing group (3.2 per 1000 person-years) and the high-stable group (2.7 per 1000 person-years) than in the normal-stable group (1.9 per 1000 person-years). Additional file 1: Table S4 shows the baseline characteristics grouped by sex and haemoglobin trajectory type.

**Table 1:** Baseline characteristics of the study population by haemoglobin trajectory groups

Variables	Haemoglobin trajectory classes			
	High-stable (*N* = 5395)	Normal-stable (*N* = 5310)	Decreasing (*N* = 726)	*p-value*
Age at entry, year	34.4 (7.2) *	33.7 (7.1)	33.8 (6.6)	< 0.001
Men, n (%)	3146 (58.3) *	3234 (60.9)	169 (23.3) *	< 0.001
BMI, kg/m^2^	24.1 (3.6) *	23.5 (3.6)	22.6 (3.2) *	< 0.001
Hypertension, n (%)	1473 (27.3) *	1050 (19.8)	109 (15.0) *	< 0.001
Dyslipidemia, n (%)	1983 (49.0) *	1604 (42.1)	189 (39.6)	< 0.001
Diabetes mellitus, n (%)	153 (2.8) *	100 (1.9)	20 (2.8)	0.004
GFR, mL/min per 1.73 m^2^	95.8 (14.4) *	98.0 (14.5)	96.2 (15.2)	< 0.001
Smoker, n (%)	835 (15.5) *	688 (13.0)	38 (5.2) *	< 0.001
Drinker, n (%)	1065 (19.7)	966 (18.2)	53 (7.3) *	< 0.001
Haemoglobin, g/L				
Baseline level	149.3 (13.0) *	138.8 (13.0)	119.3 (24.7) *	< 0.001
Mean level	149.0 (12.2) *	138.1 (12.2)	117.0 (21.2) *	< 0.001
Minimum level	143.5 (12.9) *	132.2 (13.4)	103.3 (21.9) *	< 0.001
Maximum level	154.4 (12.5) *	143.7 (12.2)	130.0 (22.4) *	< 0.001
Age at stroke, year	46.0 (5.6)	45.3 (5.9)	45.0 (5.3)	0.707
Median follow-up years	4.5 (1.1–12.4)	4.4 (1.1–12.7)	4.8 (1.7–11)	0.208
Stroke incidence density, per 1000 person-years	2.7 *	1.9	3.2	0.046

Data are presented as mean (SD), median (range) or percentage. *P* values were calculated from the comparison between 3 identified trajectory classes

* Compared with the Normal-stable class: *P* < 0.05

Abbreviations: *BMI* body mass index, *GFR* glomerular filtration rate

Table 2 presents the hazard ratios (HRs) and 95% confidence intervals (95% CIs) of stroke incidence according to haemoglobin trajectory type for the total population, as well as for men and women individually. Compared to the reference (normal-stable) group, the unadjusted HRs (95% CI) were 1.85 (0.98, 3.49) and 1.53 (1.07, 2.20) for the decreasing group and high-stable group, respectively. Adjusting for covariates of age at entry, sex, eGFR, BMI, smoking, alcohol consumption, hypertension, dyslipidaemia, and diabetes mellitus, the decreasing group had a 2.94-fold (95% CI 1.22 to 7.06) higher risk of stroke than the normal-stable group. The adjusted HR for the high-stable group was not statistically significant (HR = 1.23; 0.77, 1.97; *P* = 0.394). In the unadjusted model, men showed similar results, and the hazard ratios were 2.99 (1.07, 8.36) and 1.71 (1.14, 2.58) for the decreasing group and the high-stable group, respectively. Only the decreasing group was significant (HR = 4.12, 95% CI 1.50 to 11.28) after adjusting for potential confounders. None of the effects were significant for women in either the unadjusted or the adjusted model.

**Table 2:** Hazard ratios (HR) and 95% confidence interval (CI) of haemoglobin trajectory classes on incident stroke in the total, men and women

	Model 1^†^		Model 2^‡^	
	HR (95% CI)	*P*-value	HR (95% CI)	*P*-value
Total				
Normal-stable	Reference		Reference	
High-stable, HR (95% CI)	1.53 (1.07, 2.20)	0.020	1.23 (0.77, 1.97)	0.394
Decreasing, HR (95% CI)	1.85 (0.98, 3.49)	0.056	2.94 (1.22, 7.06)	0.016
Men				
Normal-stable	Reference		Reference	
High-stable, HR (95% CI)	1.71 (1.14, 2.58)	0.010	1.26 (0.75, 2.11)	0.376
Decreasing, HR (95% CI)	2.99 (1.07, 8.36)	0.037	4.12 (1.50, 11.28)	0.006
Women				
Normal-stable	Reference		Reference	
High-stable, HR (95% CI)	1.15 (0.52, 2.56)	0.724	1.08 (0.34, 3.49)	0.893
Decreasing, HR (95% CI)	2.43 (0.96, 6.16)	0.061	1.66 (0.34, 8.19)	0.534

*HR* hazard ratio, *CI* confidence interval;

† Unadjusted model

‡ Adjusting for baseline age, sex (only for total), smoker, drinker, GFR, BMI, diabetes mellitus, hypertension, and dyslipidemia

## Discussion

In this longitudinal study, we identified 3 distinct long-term haemoglobin concentration trajectory types in a large sample of 11,431 Chinese adults aged 20 to 48 years old. The long-term longitudinal pattern of haemoglobin were robustly characterized as high-stable, normal-stable and decreasing with the method we applied. A sex difference was observed in the trajectory analysis. Individuals with a decreasing haemoglobin trajectory had an almost three-fold increased risk of stroke, and the high-stable group comprised the most participants with a high number of traditional risk factors among the total population. After stratification by sex, these effects were not significant in women. The results from this study provide more insights into the mechanisms of haemoglobin concentration and the risk of stroke; specifically, the approximately three-fold increased risk of stroke in the decreasing trajectory group should be emphasized. These findings could help identify healthy adults with a risk of stroke in early life, and thus guide early prevention strategies.

The trajectories we identified in this study extend our understanding of the long-term changes in haemoglobin in young- to middle-aged healthy adults. Peterson et al. reported 6 distinct trajectories of haemoglobin in patients after hospitalization for heart failure and showed that a persistent decline and anaemia were associated with an increased mortality risk [11]. Our results showed the same trajectory type associated with an increased risk of stroke. However, we found no evidence of this effect in women. We also found that haemoglobin level may not only differ between sexes but also differ with age.

In the study population, the participants in the decreasing trajectory group appeared to be the healthiest at baseline, but the individuals with a decreasing haemoglobin trajectory pattern had the highest risk of developing stroke. Notably, the average maximum haemoglobin (130.0 g/L) in the decreasing group was near the limit of the World Health Organization’s definition of anaemia (males, 130 g/L; females, 120 g/L) [18]. The concentration of haemoglobin consistently decreased with age. Anaemia or low haemoglobin was associated with an increased risk of cardiac implications [19]. Studies have described several pathological mechanisms that could explain the association between low haemoglobin and the risk of stroke. Low haemoglobin levels lead to cerebral blood insufficiency, which reduces the oxygen-carrying capacity and results in distal-field tissue ischaemic injury [20]. Moreover, iron-deficiency anaemia was reportedly a risk factor for stroke [21]. Our study suggested that the haemoglobin-stroke association existed in men but not in women. This difference may be explained by the protective effect of oestrogen in women, as the stroke incidence was lower in premenopausal women than in men [8]. Sex hormones regulate vascular oxidative stress, which is believed to play an important role in the aetiology of stroke [22]. In addition, endogenous oestrogen levels were positively correlated with a substantial increase in cerebral blood flow [23].

It should be noted that even though the high-stable group had the highest haemoglobin concentration, the hazard ratio was less than that in the decreasing group. A prospective study of 808,143 young Korean women reported that a high haemoglobin concentration increased the risk of stroke [24]. Another study from community-living adults also showed that low or high haemoglobin was associated with an increased risk of stroke [4]. However, our study showed a non-significant positive association between a high haemoglobin concentration and the risk of stroke. The difference may be explained by the different study methods. The method we applied in this study differed from those in common practice, which only consider baseline haemoglobin levels or define a threshold; instead, this study focused on the development of haemoglobin trajectory. In addition, we identified distinct mutually exclusive haemoglobin trajectories by age that were not captured by conventional analytical approaches. Treatment aimed to increase the haemoglobin level has been shown to double the risk of stroke [6], but the responsible factor, such as changes in the haemoglobin level or increased erythropoiesis-stimulating agent use, for the increased risk is still unknown [25]. The decreasing group had the highest haemoglobin standard deviation in our study, which may indicate that a change in the haemoglobin level itself increases the risk of stroke. The mechanism of these results should be studied systematically in the future. Early prevention should focus on patients who are seemingly healthy but present consistently decreasing haemoglobin levels.

We observed an increased proportion of traditional risk factors of stroke at baseline in the high-stable group. After adjusting for confounders, the hazard ratio for the high-stable group was no longer significant, as the effect was attenuated by other traditional risk factors in the adjusted model. This indicates that haemoglobin in the high-stable group was not an independent risk factor for incident stroke. Kubo et al. [26] and Qin et al. [27] reported that anaemia was associated with underweight. The association of haemoglobin with hypertension, cholesterol, eGFR, and smoking was also studied [28–31]. However, the exact role of haemoglobin in the pathway of those risk factors and stroke is still unknown.

The strengths of our study are the large population cohort and the repeated haemoglobin measurements over a long follow-up period. We acknowledge that our study has limitations from a cohort perspective. The reporting of a stroke event was derived from the available medical insurance and hospital-linked databases, so some disease incidence data may have been missing or reported late. As the study population was healthy adults, the incidence of stroke reported here was lower than that in previous studies from China, where the incidence of first-ever stroke ranged from 116 to 219 per 100,000 people per year [32]. To address this issue, we extended the stroke events report to 2017 to account for delayed reports of stroke incidence, and we believe that the reporting scheme did not affect our results. In addition, detailed stroke data were not available, so the study considered any type of stroke.

## Conclusions

A decreasing haemoglobin trajectory was significantly associated with a nearly three-fold increased risk of stroke compared to the normal-stable trajectory. Although individuals seemed healthy at baseline, a persistent decrease in haemoglobin increased the risk of stroke in men. This information can be used to identify patients at risk of stroke in early life and therefore guide early prevention strategies. Our findings provide new insights into identifying adults with a risk of stroke at an early age; however, the mechanisms need to be explored systematically in the future.

## Supplementary information

**Additional file 1. **Supplementary information for Trajectories of Haemoglobin and incident stroke risk: a longitudinal cohort study. **Figure S1.** Flowchart showing numbers of patients excluded from the analysis. **Table S1.** Baseline characteristics of participants included and excluded (< 3 haemoglobin assessments). **Table S2.** Latent Class Growth Mixture models (LCGMM) results of model fitting process. **Table S3.** Parameter estimates for the best fitting 3-class cubic latent class growth mixture model fitted to the haemoglobin data. **Table S4.** The baseline characteristics of the study population by haemoglobin trajectory classes and sex.

## Data Availability

The data that supporting the findings of this study are available from the corresponding authors upon request.

## Abbreviations

BIC: Bayesian information criterion
BMI: Body mass index
CI: Confidence interval
CKD-EPI: Chronic Kidney Disease Epidemiology Collaboration
eGFR: Estimated glomerular filtration rate
HR: Hazard ratio
ICD-10: The international classification of diseases, 10th revision
LCGMM: Latent class growth mixed model
