# Supplementary material for: Trajectories of Haemoglobin and incident stroke risk: a longitudinal cohort study
Source: BMC Public Health. 2019 Oct 28;19:1395. doi: 10.1186/s12889-019-7752-7 (PMC6819541; doi:10.1186/s12889-019-7752-7)
Supplement: Supplementary file 1 — Additional file 1. Supplementary information for Trajectories of Haemoglobin and incident stroke risk: a longitudinal cohort study. Figure S1. Flowchart showing numbers of patients excluded from the analysis. Table S1. Baseline characteristics of participants included and excluded (< 3 haemoglobin assessments). Table S2. Latent Class Growth Mixture models (LCGMM) results of model fitting process. Table S3. Parameter estimates for the best fitting 3-class cubic latent class growth mixture model fitted to the haemoglobin data. Table S4. The baseline characteristics of the study population by haemoglobin trajectory classes and sex. [file 12889_2019_7752_MOESM1_ESM.docx]

**Additional file 1**

Supplementary information for **Trajectories of Haemoglobin and Incident Stroke Risk: A Longitudinal Cohort Study**

**Figure S1.** Flowchart showing numbers of patients excluded from the analysis.

**
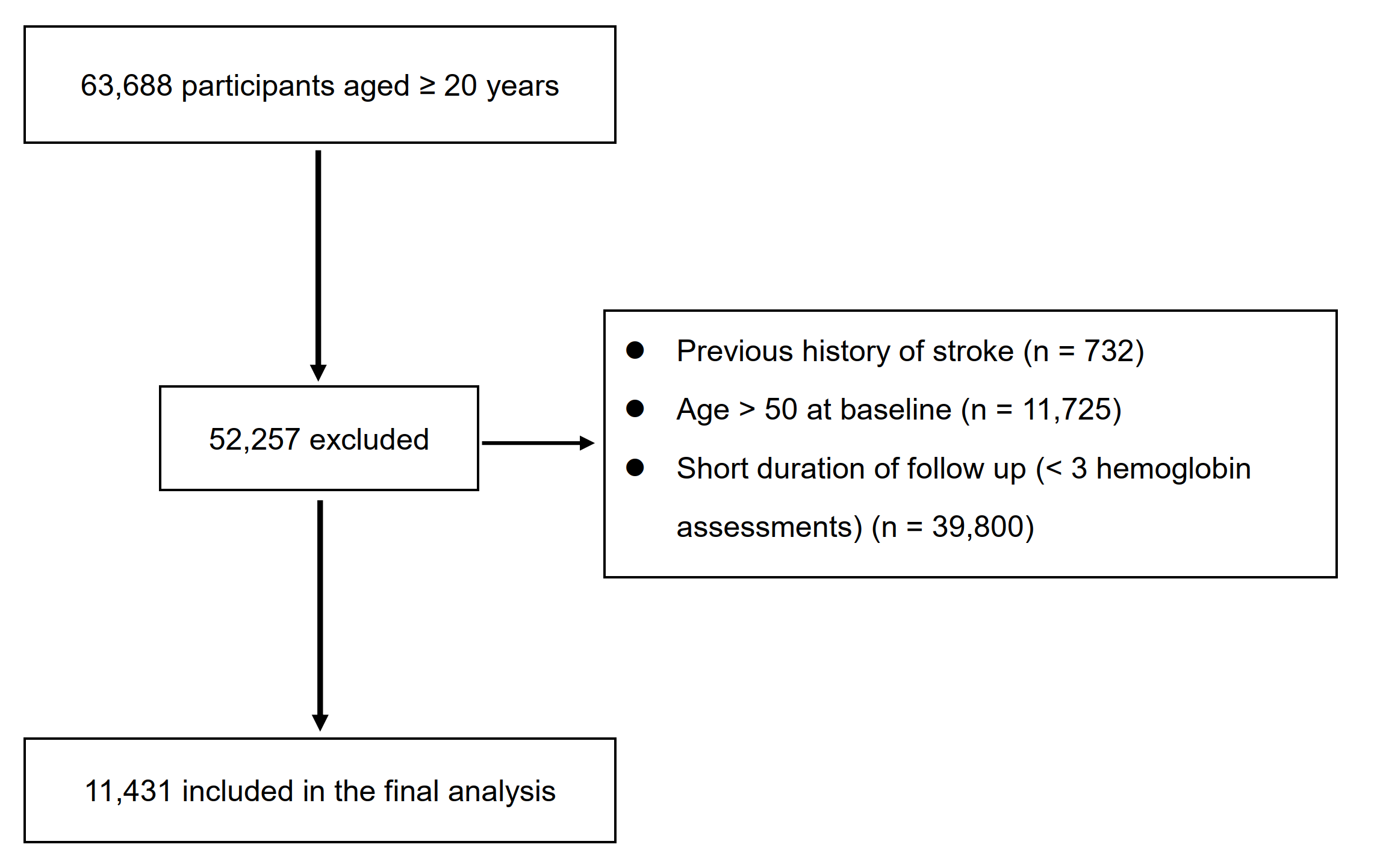
**

**Table S1.** Baseline characteristics of participants included and excluded (< 3 haemoglobin assessments).

| **Variable** | **Excluded (N=52257)** | **Included (N=11431)** |
| --- | --- | --- |
| Age at entry, year | 34.4 (8.6) | 34.0 (7.1) |
| Men, n (%) | 23873 (60.0) | 6549 (57.3) |
| Baseline haemoglobin, g/L | 143.0 (16.4) | 142.5 (16.1) |
| GFR, mL/min per 1.73 m^2^ | 93.6 (12.9) | 96.8 (14.5) |
| BMI, kg/m^2^ | 24.3 (3.9) | 23.7 (3.6) |
| Smoker, n (%) | 3838 (9.6) | 1561 (13.7) |
| Drinker, n (%) | 4595 (11.5) | 2084 (18.2) |
| Hypertension, n (%) | 6814 (17.1) | 2632 (23.0) |
| Dyslipidemia, n (%) | 11859 (42.1) | 3776 (45.3) |
| Diabetes mellitus, n (%) | 1406 (3.5) | 273 (2.4) |

Data are presented as mean (SD), median (range) or percentage.

Abbreviations: BMI, body mass index; GFR, glomerular filtration rate

**Table S2.** Latent Class Growth Mixture models (LCGMM) results of model fitting process

| **No. of**  **latent classes** | **Polynomial**  **degree** | **Log-Lik** | **BIC** | **% Participants per class** | **Mean posterior probabilities** | **Posterior probabilities > 0.7 (%)** |
| --- | --- | --- | --- | --- | --- | --- |
| 1 | Linear | -168095.5 | 336265.76 |  |  |  |
|  | Quadratic | -167804.68 | 335730.84 |  |  |  |
|  | Cubic | -167754.04 | 335685.62 |  |  |  |
| 2 | Linear | -166933.8 | 333979.73 | 3.46/96.54 | 0.89/0.98 | 85.06/99.29 |
|  | Quadratic | -166549.45 | 333267.1 | 4.58/95.42 | 0.88/0.97 | 82.82/98.59 |
|  | Cubic | -166449.69 | 333132.99 | 4.79/95.21 | 0.88/0.97 | 80.66/98.54 |
| 3 | Linear | -166624.83 | 333399.16 | 3.79/0.97/95.24 | 0.82/0.88/0.96 | 74.36/81.08/98.14 |
|  | Quadratic | -166433.78 | 333082.47 | 5.76/47.63/46.61 | 0.87/0.76/0.76 | 78.27/66.04/64.53 |
|  | **Cubic** | **-166316.94** | **332923.54** | **6.35/47.2/46.45** | **0.87/0.77/0.76** | **78.1/68.8/66.65** |
| 4 | Linear | -166558.91 | 333304.7 | 0.75/1.45/94.76/3.04 | 0.88/0.74/0.94/0.73 | 79.07/58.43/97.4/56.77 |
|  | Quadratic | -166283.85 | 332829.34 | 0.82/43.64/48.98/6.55 | 0.82/0.74/0.73/0.79 | 74.47/62.8/61.23/67.82 |
|  | Cubic | -166174.55 | 332694.84 | 6.73/26.8/20.92/45.56 | 0.87/0.77/0.79/0.7 | 79.97/68.82/69.85/55.97 |
| 5 | Linear | -166534.16 | 333292.57 | 0.8/1.33/43.64/50.23/4 | 0.86/0.7/0.72/0.69/0.73 | 81.32/51.97/55.3/52.56/56.46 |
|  | Quadratic | -166202.9 | 332714.15 | 0.71/6.75/28.21/44.9/19.43 | 0.81/0.8/0.72/0.67/0.75 | 74.07/69.69/59.22/42.91/61.82 |
|  | Cubic | -166077.68 | 332557.15 | 6.27/18.21/33.93/31.42/10.17 | 0.88/0.76/0.68/0.72/0.77 | 81.73/64.07/46.73/57.02/66.44 |

Reported are: the number of latent class considered, the polynomial form of the model, the maximum Log-Likelihood (Log-Lik), the Bayesian information Criterion (BIC), the posteriori classification of subjects in each class (%), the mean of posterior probabilities in each latent class, and the % of subjects classified in each class with a posterior probability above 0.7. The best fitting model is highlighted in bold characters.

**Table S3.** Parameter estimates for the best fitting 3-class cubic latent class growth mixture model fitted to the haemoglobin data.

| **Polynomial term** | **Class** | **Coefficient** | **Standard error** | **Wald** | ***p*-value** |
| --- | --- | --- | --- | --- | --- |
| *Intercept* | High-stable | 133.91 | 0.19 | 710.37 | <0.001 |
|  | Normal-stable | 124.3 | 0.2 | 616.37 | <0.001 |
|  | Decreasing | 115.87 | 0.96 | 120.17 | <0.001 |
| *age* | High-stable | -0.38 | 0.28 | -1.34 | 0.182 |
|  | Normal-stable | -0.68 | 0.38 | -1.78 | 0.076 |
|  | Decreasing | -10.78 | 1.56 | -6.91 | <0.001 |
| *age^2* | High-stable | 0.71 | 0.23 | 3.12 | 0.002 |
|  | Normal-stable | 1.51 | 0.24 | 6.18 | <0.001 |
|  | Decreasing | 2.4 | 1.13 | 2.13 | 0.033 |
| *age^3* | High-stable | -0.07 | 0.18 | -0.42 | 0.671 |
|  | Normal-stable | -0.28 | 0.29 | -0.97 | 0.332 |
|  | Decreasing | 2.36 | 1.25 | 1.88 | 0.060 |
| *sex* |  | 24.14 | 0.2 | 123.39 | <0.001 |
| *sex*age* |  | 0.17 | 0.4 | 0.42 | 0.678 |
| *sex*age^2* |  | -1.26 | 0.24 | -5.33 | <0.001 |
| *sex*age^3* |  | -0.45 | 0.28 | -1.6 | 0.109 |

**Table S4.** The baseline characteristics of the study population by haemoglobin trajectory classes and sex

|  | **Men** | | | |  | **Women** | | | |
| --- | --- | --- | --- | --- | --- | --- | --- | --- | --- |
| **Variables** | **High-stable**  **(N = 3146)** | **Normal-stable**  **(N = 3234)** | **Decreasing**  **(N = 169)** | ***P*-value** |  | **High-stable**  **(N = 2249)** | **Normal-stable**  **(N = 2076)** | **Decreasing**  **(N = 557)** | ***P*-value** |
| Age at entry, year | 35.0 (7.1) | 34.4 (6.9) | 33.4 (6.8) | <0.001 |  | 33.6 (7.4) | 32.6 (7.1) | 33.9 (6.6) | <0.001 |
| BMI, kg/m^2^ | 25.5 (3.4) | 24.7 (3.5) | 24.7 (3.5) | <0.001 |  | 22.1 (3.1) | 21.7 (2.9) | 22.0 (2.8) | <0.001 |
| Hypertension, n (%) | 1211 (38.5) | 921 (28.5) | 58 (34.3) | <0.001 |  | 262 (11.6) | 129 (6.2) | 51 (9.2) | <0.001 |
| Dyslipidemia, n (%) | 1374 (54.7) | 1157 (46.1) | 55 (49.1) | <0.001 |  | 609 (39.6) | 447 (34.3) | 134 (36.7) | 0.014 |
| Diabetes mellitus, n (%) | 119 (3.8) | 84 (2.6) | 9 (5.3) | 0.008 |  | 34 (1.5) | 16 (0.8) | 11 (2.0) | 0.024 |
| GFR, mL/min per 1.73 m^2^ | 97.9 (14.1) | 99.6 (14.2) | 102.1 (15.4) | <0.001 |  | 92.4 (14.4) | 94.9 (14.6) | 94.1 (14.6) | <0.001 |
| Smoker, n (%) | 832 (26.4) | 688 (21.3) | 36 (21.3) | <0.001 |  | 3 (0.1) | 0 (0.0) | 2 (0.4) | 0.052 |
| Drinker, n (%) | 1058 (33.6) | 963 (29.8) | 50 (29.6) | 0.004 |  | 7 (0.3) | 3 (0.1) | 3 (0.5) | 0.236 |
| Haemoglobin, g/L |  |  |  |  |  |  |  |  |  |
| Baseline level | 158.9 (6.3) | 147.7 (6.4) | 142.7 (22.3) | <0.001 |  | 135.9 (6.3) | 124.9 (6.9) | 112.2 (20.7) | <0.001 |
| Mean level | 158.7 (4.4) | 147.2 (4.2) | 141.5 (19.2) | <0.001 |  | 135.4 (4.2) | 123.8 (4.4) | 109.5 (15.3) | <0.001 |
| Minimum level | 153.3 (5.4) | 141.8 (5.6) | 126.3 (24.6) | <0.001 |  | 129.8 (5.6) | 117.3 (6.9) | 96.2 (15.1) | <0.001 |
| Maximum level | 163.8 (5.3) | 152.4 (5.1) | 153.0 (19.0) | <0.001 |  | 141.1 (5.8) | 130.0 (5.6) | 123.0 (18.3) | <0.001 |
| Age at stroke, year | 45.4 (5.7) | 45.3 (5.9) | 47.5 (2.1) | 0.764 |  | 48.7 (3.9) | 45.1 (6.1) | 43.8 (6.0) | 0.095 |
| Median follow-up years | 4.8 (1.1, 12.4) | 4.8 (1.1, 12.7) | 4.1 (1.7, 10.4) | 0.729 |  | 4.2 (1.3, 11.3) | 4.1 (1.5, 11.9) | 4.8 (1.7, 11.3) | 0.017 |
| Stroke incidence density,  per 1000 person-years | 3.7 | 2.3 | 4.9 | 0.014 |  | 1.2 | 1.2 | 2.7 | 0.148 |

Data are presented as mean (SD), median (range) or percentage. P-values were calculated from the comparison between 3 identiﬁed classes. Abbreviations: BMI, body mass index; GFR, glomerular filtration rate
